# Expression profiling to predict the clinical behaviour of ovarian cancer fails independent evaluation

**DOI:** 10.1186/1471-2407-8-18

**Published:** 2008-01-22

**Authors:** Olivier Gevaert, Frank De Smet, Toon Van Gorp, Nathalie Pochet, Kristof Engelen, Frederic Amant, Bart De Moor, Dirk Timmerman, Ignace Vergote

**Affiliations:** 1 Department of Electrical Engineering ESAT-SCD-Sista, Katholieke Universiteit Leuven, Kasteelpark Arenberg 10, 3001 Leuven, Belgium; 2 Medical Direction, National Alliance of Christian Sickness Funds, Haachtsesteenweg 579, 1031 Brussel, Belgium; 3 Department of Obstetrics and Gynaecology, UZ Leuven, Campus Gasthuisberg, Katholieke Universiteit Leuven, Herestraat 49, 3000 Leuven, Belgium; 4 Broad Institute of Harvard and MIT, 7 Cambridge Center, Cambridge, MA 02142, USA; 5 FAS Center for Systems Biology, Harvard University, 7 Divinity Avenue, Cambridge, MA 02138, USA; 6 Department of Microbial and Molecular systems CMPG, Katholieke Universiteit Leuven, Kasteelpark Arenberg 20, 3001 Leuven, Belgium

## Abstract

**Background:**

In a previously published pilot study we explored the performance of microarrays in predicting clinical behaviour of ovarian tumours. For this purpose we performed microarray analysis on 20 patients and estimated that we could predict advanced stage disease with 100% accuracy and the response to platin-based chemotherapy with 76.92% accuracy using leave-one-out cross validation techniques in combination with Least Squares Support Vector Machines (LS-SVMs).

**Methods:**

In the current study we evaluate whether tumour characteristics in an independent set of 49 patients can be predicted using the pilot data set with principal component analysis or LS-SVMs.

**Results:**

The results of the principal component analysis suggest that the gene expression data from stage I, platin-sensitive advanced stage and platin-resistant advanced stage tumours in the independent data set did not correspond to their respective classes in the pilot study. Additionally, LS-SVM models built using the data from the pilot study – although they only misclassified one of four stage I tumours and correctly classified all 45 advanced stage tumours – were not able to predict resistance to platin-based chemotherapy. Furthermore, models based on the pilot data and on previously published gene sets related to ovarian cancer outcomes, did not perform significantly better than our models.

**Conclusion:**

We discuss possible reasons for failure of the model for predicting response to platin-based chemotherapy and conclude that existing results based on gene expression patterns of ovarian tumours need to be thoroughly scrutinized before these results can be accepted to reflect the true performance of microarray technology.

## Background

Ovarian cancer ranks fifth when considering cancer mortality in women [1]. Unfortunately clinical or pathologic variables that can reliably predict recurrence in FIGO (Fédération Internationale de Gynécologie Obstétrique) stage I patients or resistance to platin-based chemotherapy in advanced stage disease (FIGO stage III or IV) are not available. The prognosis might be more optimally predicted based on gene expression analysis, since microarrays can capture tumour properties that might not be reflected in the commonly used clinical or histopathological variables at diagnosis.

Previously, we performed a pilot study consisting of microarray analysis on three groups of patients: seven stage I without recurrence, seven platin-sensitive advanced stage and six platin-resistant advanced stage ovarian tumours [2]. We investigated whether gene expression analysis can be used to distinguish between stage I and advanced stage ovarian tumours, and between platin-sensitive and platin-resistant ovarian tumours. The results showed that a considerable number of genes were differentially expressed between the different tumour classes. This was confirmed by principal component analysis (PCA) where the distinction between the three tumour classes was visualised. A least squares support vector machine (LS-SVM) analysis showed that the estimated classification performance was 100% for the distinction between stage I and advanced stage disease, and 76.92% for the distinction between platin-sensitive and platin-resistant disease when using a leave-one-out approach. These results indicated that gene expression analysis could be appropriate to predict prognosis of ovarian tumours. However, since leave-one-out cross validation can overestimate the performance of a model, an independent evaluation is needed to have an unbiased estimate of the generalization capacity.

In the current study, we describe results of an independent evaluation of models for predicting disease stage and response to platin-based chemotherapy built on the data of the pilot. Our goal was to evaluate whether an independent study could confirm the applicability of microarrays for the clinical management of ovarian cancer. This independent evaluation was carried out on a set of 49 new tumour samples which were subjected to the same experimental protocol. This data set was used as a test set to estimate the performance when predicting the difference between stage I and advanced stage disease, and between platin-sensitive and platin-resistant disease using models trained on the pilot data set. After presenting the results, we discuss the generalization performance on this independent data set and compare with models based on previously published gene sets.

## Methods

### Tumour characteristics

Tissue collection and analysis were approved by the local ethical committee. After obtaining informed consent, tumour biopsies were sampled and immediately frozen in liquid nitrogen during primary surgery and were taken from three groups of patients: 4 from patients with stage I disease, 30 from patients with platin-sensitive advanced stage disease and 15 from patients with platin-resistant advanced stage disease [3]. In this study, similarly as in the pilot study, we will refer to these three groups as: I, A_s _and A_r _respectively. The patient and tumour characteristics are shown in table 1.

**Table 1:** Tumour characteristics. Clinical information of the tumour samples in the independent data set

	Class Ar (n = 15)	Class As (n = 30)	Class I (n = 4)
Mean Age (range), years	61.8	61.3	49
*Histologic type*			
Serous	14	29	1
Endometrioid	1	-	2
Mucinous	-	-	1
Mixed carcinoma	-	1	-
*FIGO stage*			
I	-	-	4
III	9	28	-
IV	6	2	-
*Differentiation grade*			
Grade 1	-	1	1
Grade 2	5	7	2
Grade 3	10	22	1
*Operation*			
Primary surgery	6	22	4
Interval surgery after three courses of chemotherapy	3	8	-
Diagnostic biopsy, no surgery	6	-	-
*Residual tumour load after surgery*			
0 cm	8	24	4
0–1 cm	-	1	-
1–2 cm	-	4	-
> 2 cm	7	1	-
*Time to progression after first-line chemotherapy*			
< 6 months	15	-	-
6–12 months	-	-	1
> 12 months	-	22	2
No recurrence	-	8	1
*Current status*			
No evidence of disease	-	8	1
Alive with evidence of disease	1	8	1
Died of disease	14	14	2
*Median follow-up, months*	16	35	18

### Microarray procedures

Microarray procedures were similar to our pilot study [2]. Briefly, each tumour in the independent data set was hybridized twice (dye-swap) against the same common reference pool from the pilot study on an array containing 21.372 probes enriched for genes related to ovarian cancer. From each patient, mRNA was amplified and labelled with Cy3 and Cy5, according to Puskas and collaborators [4]. All protocols can be downloaded from ArrayExpress [5]. Microarray data and information recommended by the MIAMI (Minimum Information About a MIcroarray experiment) guidelines can be found on the ArrayExpress website [6] (Accession number E-MEXP-995 for the independent data set and E-MEXP-979 for the pilot data).

### Microarray data analysis

The gene expression data were analysed using MATLAB 7 (R2006b). Pre-processing was done similarly as in our pilot study. Briefly, each microarray in the independent data set was analysed separately in the following order: the intensities were background-corrected, log-transformed and finally normalised using the intensity dependent Lowess fit procedure. The mean of the replicate and normalised log ratios was used as a measure for expression. After pre-processing, first PCA and secondly LS-SVM were used to analyse the data. PCA was used for visualisation of the data while LS-SVMs were used for building classification models. A p-value is considered statistically significant if smaller than 0.05. All statistical tests were two-sided unless mentioned otherwise. Exact bionomial confidence intervals were calculated using SAS 9.1.3 statistical software.

### PCA

**Figure 1:**
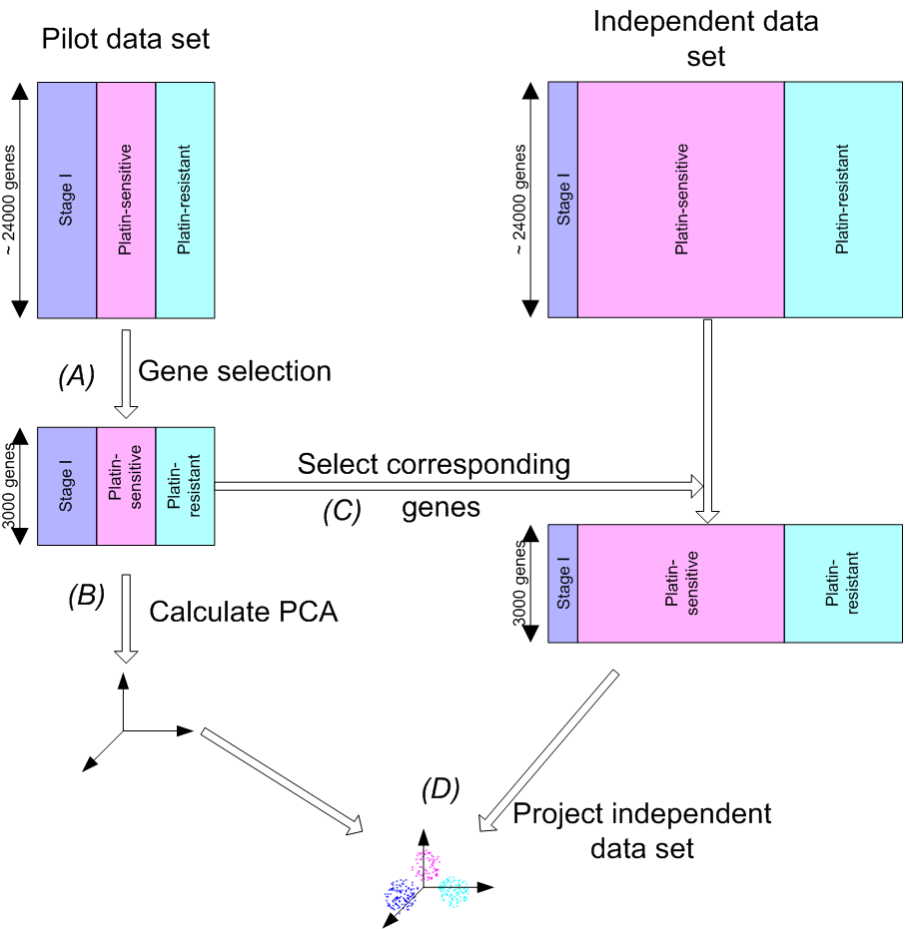
**Principal Component Analysis (PCA)**. Both the pilot and independent data set are shown. First, (A) gene selection is done using a Kruskal Wallis test. Then (B) PCA analysis is performed on this reduced pilot data set. Next (C), the corresponding gene expression values are selected in the independent data set and finally (D) this reduced independent data set is projected on the three largest principal components calculated only on the pilot data set.

The procedure followed during PCA analysis can be found in Figure 1. This figure schematically shows the different steps involving the pilot and the independent data set. First, we rank the genes according to their differential expression between the three classes (Kruskal Wallis test) the pilot data and the top 3000 genes were selected. Then PCA analysis was performed on the reduced pilot data set and the three largest principal components were selected (i.e., the directions associated with the largest eigenvalues). Finally, we used the gene expression values from the independent data set corresponding to the 3000 genes that were previously selected in the pilot data set and projected this reduced independent data set in the space defined by the three largest principal components in the pilot data. Finally, the 3000 corresponding gene expression values were selected in the independent data set and the reduced independent data set was projected in this space.

### LS-SVMs

**Figure 2:**
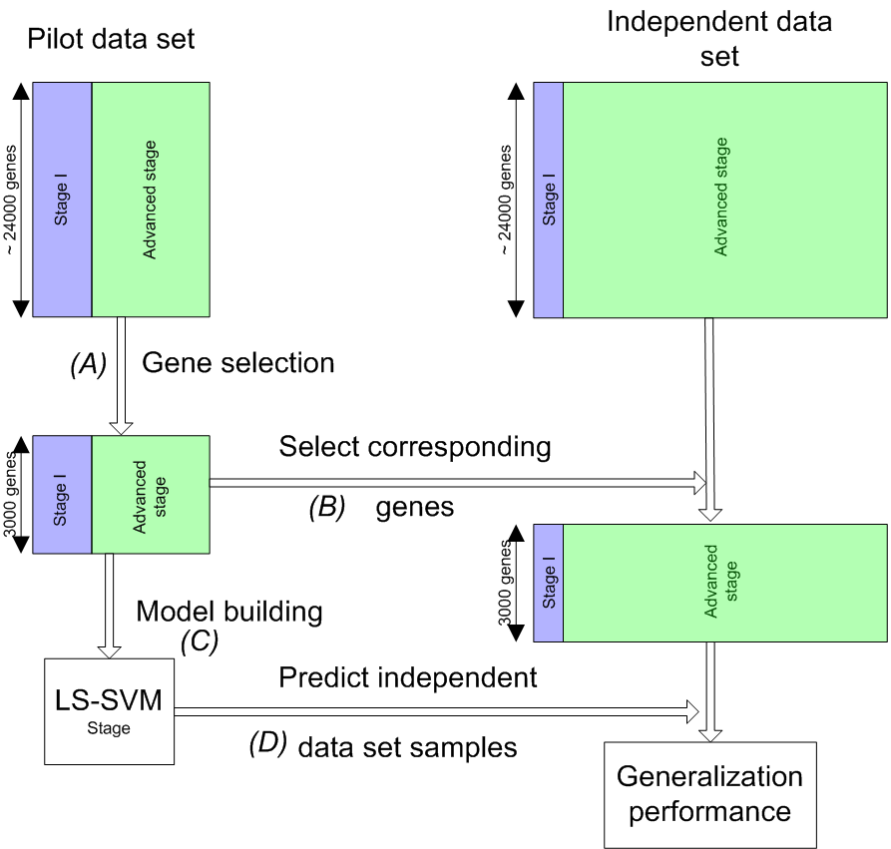
**Model building procedure**. LS-SVM model building procedure for disease stage, the model building procedure for response to platin-based chemotherapy is similar. First (A), the 3000 genes with the largest degree of differential expression between two classes in the pilot study are selected. Next (B), the corresponding gene expression values are selected in the independent data set. Subsequently, (C) an LS-SVM model is built using only the pilot data. Finally, (D) this model is used to predict the class of the samples in the independent data set which gives an estimate of the generalization performance.

Next, we used the pilot data set to build an LS-SVM to predict disease stage and an LS-SVM to predict the response to platin-based chemotherapy (MATLAB scripts were downloaded from LS-SVMlab version 1.5 [7,8]). In the pilot study, an RBF kernel did not improve results therefore in all subsequent analysis a linear kernel was used. Figure 2 shows the different steps in this analysis which consists of the same steps for both two-class classification problems. First, the genes were ranked according to the differential expression between two classes using only the pilot study data and the top 3000 genes in this ranking were selected (Wilcoxon rank sum test). Next, the corresponding gene expression values were selected in the independent data set. Subsequently, an LS-SVM with linear kernel was trained using the reduced pilot data and applied to predict the class of the samples in the independent data set. This results in a estimate of the generalization performance of a model built only on the pilot study data for both classification problems.

### Comparison with other profiles

**Figure 3:**
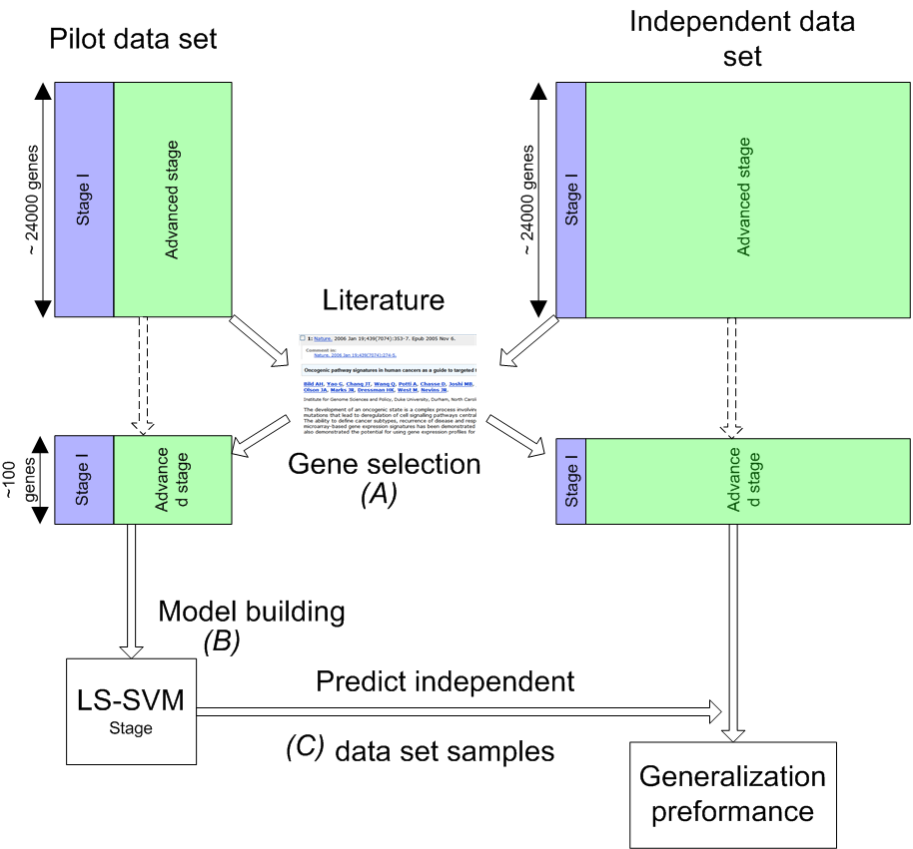
**Model building procedure published gene sets**. Model building procedure for testing the performance of published gene sets that predict ovarian cancer outcomes. First (A), the gene set is extracted from the literature and the corresponding genes are selected in both the pilot and independent data set. Subsequently, (B) an LS-SVM model is built using only the pilot data and finally, (C) this model is used to predict the class of the samples in the independent data set which gives an estimate of the generalization performance of a gene set.

To assess the performance of models based on our data we compared them with the performance of models based on published gene sets that predict a broad range of outcomes in ovarian cancer. It is difficult to directly apply the published models on our data since multiple different microarray platforms (e.g. one channel Affymetrix microarrays(Uv95Av2, HumanGeneFl, U133A) or two-channel custom arrays (cDNA)) have been used to derive these gene sets. Therefore we adopted the strategy visualized in Figure 3. First, the gene set is extracted from the literature and, if not already done, the genes were translated to HUGO (Human Genome Organization) gene symbols. Then, we extracted, in both the pilot and independent data set, the genes corresponding to the HUGO gene set from the literature. Subsequently, our model building strategy proceeds as previously described (see Figure 3). We used gene sets related to the response on platin-based chemotherapy [9-11], gene sets related to survival in epithelial ovarian cancer (EOC) [12] or in advanced stage serous EOC [13,14], gene sets discriminating between the major histological types (serous, mucinous, clear cell and endometrioid) [15,16], gene sets distinguishing between normal ovarian tissue and disease [17,18], gene sets discriminating between low malignant potential or borderline disease and invasive disease [19], gene sets differentiating between ovarian cancer tissue and metastatic tissue [20] and a gene set predicting the presence of disease at second look surgery [21]. These gene sets where constructed based on affymetrix microarrays (HuGeneFl, U95 set, U95Av2, U133A), different cDNA microarrays or HPLC (High Performance Liquid Chromatography) followed by ESI-TOF (Electrospray Ionization Time of Flight) mass spectrometry.

## Results

**Figure 4:**
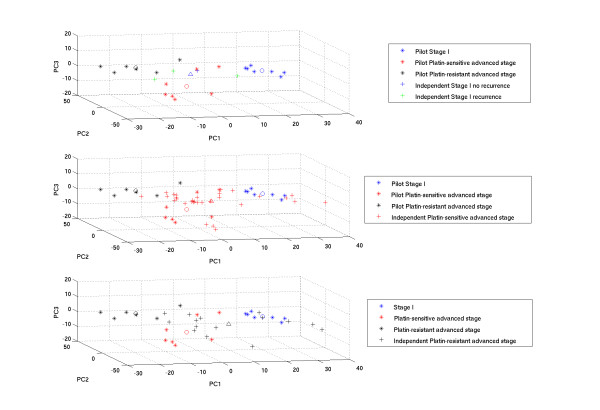
**Results of the Principal Component Analysis (PCA)**. Visualization of the three principal component directions corresponding to the largest variation in the pilot data after selection of the 3000 genes with the largest degree of differential expression (Kruskal-Wallis) and projection of the independent data set onto these principal components. * = individual pilot sample, + = individual independent data set sample, o = mean expression in each class in the pilot data, Δ = mean expression of the projected independent data set class; blue = stage I without recurrence; green = stage I with recurrence; red = platin-sensitive advanced stage; black = platin-resistant advanced stage. **Top panel)** projection of the stage I independent data set samples, **Middle** panel) projection of the platin-sensitive advanced stage independent data set samples, **Bottom panel**) projection of the platin-resistant advanced stage independent data set samples.

In this study we describe the results of the evaluation of models developed based on the data from our previously published pilot study [2] using PCA analysis or LS-SVMs on independently gathered microarray data. Note that all stage I patients in the pilot study had ovarian tumours without recurrence while in the current study population the four patients with stage I disease consist of 3 stage I tumours with recurrence and 1 stage I tumour without recurrence. Figure 4 shows the results of the PCA analysis. This figure visualises the projection of the patients from the independent data set belonging to the stage I, platin-sensitive and platin-resistant group onto the three principal component directions calculated based on the pilot study data. For all three groups, the data are scattered around the origin which indicates that the principal components computed based on the pilot data were not able to reproduce the three classes in the independent data set. Additionally, we did not observe a clear distinction between the stage I patients with and without recurrence (see Figure 4, top panel).

Secondly, we used LS-SVMs to assess if a supervised classification model can discriminate between the stage I and advanced stage disease, and between platin-sensitive and platin resistant disease. This resulted in a classification accuracy of 97.96% (CI 19%–99%) for the distinction between stage I and advanced stage disease which corresponds to one stage I tumour out of four that was classified as an advanced stage tumour. Next, a classification accuracy of 51.11% was obtained for the distinction between platin-sensitive and platin-resistant disease. This corresponds to five platin-resistant and eighteen platin-sensitive tumours that were misclassified, corresponding to a sensitivity of 67% (CI 38%–0.88%) and specificity of 40% (CI 23%–0.59%) when considering a platin resistant patient as a positive

Table 2 shows the accuracy on the independent data set for predicting stage and platin sensitivity of the models based on the pilot data and previously published gene sets. Most gene sets are able to predict ovarian cancer stage reliably (ranging from 87.8%–97.96%). Five profiles were less successful: Lancaster disease vs. normal (79.6%), Roberts platin sensitivity vs. platin resistance (75.5%) and both Lancaster ovarian cancer tissue vs. metastatic tissue models (71.4% and 57.14%). When focusing on the prediction of platin sensitivity, 5 of the published gene sets predicted the majority class on the independent data set resulting in 66.6% (30/45) classification accuracy. However, such a classifier has very little practical use since it predicts the same class for all independent data set samples. Finally, the Lancaster metastasis model consisting of 25 genes performed best with an accuracy of 60% corresponding to a sensitivity of 86% and specificity of 47% when considering a platin resistant patient as a positive (P-value 0.12, one sided binomial test).

**Table 2:** Comparison with published gene sets. Accuracy of all published gene set models on the independent data set both when predicting stage and platin resistance ranked by stage accuracy. Gene sets have been named after the first author of the publication followed by a description of its relationship to patient outcome. References have been used when the same first author had multiple publications

Gene set first author	Description	Stage accuracy (%)	Platin accuracy (%)
Ouellet	*low malignant potential/borderline disease vs. invasive disease: tumour tissue*	97.96	55.56
Hibbs	*Disease vs. normal or other tissues*	95.92	66.67*
Spentzos	*Residual disease vs. complete response at second look surgery*	93.88	37.78
Lu	*Disease vs. normal*	93.88	48.89
Helleman	*Platin sensitivity vs. platin resistance: differential expression*	93.88	44.44
Ouellet	*low malignant potential/borderline disease vs. invasive disease: primary cultures*	91.84	53.33
Zhu	*Clear cell vs. serous histology*	91.84	66.67*
Lancaster [14]	*Short-term vs. long-term survival*	91.84	44.44
Helleman	*Platin sensitivity vs. platin resistance: 16-gene predictive model*	91.84	46.67
Berchuck	*Short-term vs. long-term survival*	91.84	55.56
Schwartz	*Clear cell vs. other histological types*	91.84	46.67
Hartmann	*Early vs. late relapse after platin based chemotherapy*	91.84	66.67*
Spentzos	*Short-term vs. long-term survival*	87.76	66.67*
Lancaster [14]	*Disease vs. normal*	79.59	66.67*
Roberts	*Platin sensitivity vs. platin resistance*	75.51	42.22
Lancaster [20]	*Ovarian cancer tissue vs. metastatic tissue: 27-gene predictive model*	71.43	60.00#
Lancaster [20]	*Ovarian cancer tissue vs. metastatic tissue: differential expression*	57.14	66.67*

*models predicting only the majority class (platin sensitive patients) on the independent data set

#best platin model

## Discussion

Recently, several studies have investigated the use of microarrays to predict several clinically relevant outcomes of ovarian cancer [9,10,12,13,15,21]. However, the identified gene sets or developed models in these studies have not been properly evaluated on independently gathered data. Microarray technology is notorious for its low signal-to-noise ratio, suffering from many potential experimental sources of error (e.g. dye effect, print-tip effect, array effect) on top of the biological variation inherent to the samples. Moreover due to the huge number of genes (e.g. ~25.000) compared to the low number of samples (~50), overfitting models is a real danger. This occurs when models fit the training data too well and are not capable of predicting new samples. Overfitting can only be detected when using proper cross-validation techniques or independent test set analysis. Only a true independent test set – not used for determining pre-processing parameters, selection of differentially expressed genes, model building or model selection – can be used to estimate the true performance of models [22]. For example, we noticed a case of inappropriate use of a test set where this data set was used to select the best model [10,22]. This implies that the model will perform well on this particular test set but, due to the high-dimensional nature of microarray data, this performance might be impossible to reproduce on truly independent data. Moreover, a recently published review of published microarray studies that focus on cancer related outcomes showed that the most common flaw in classification studies is a biased estimation of the accuracy (present in 12 of 28 studies published in 2004 [23]). This illustrates that inappropriate evaluation of classifiers based on microarray data is a common problem when building models to predict cancer outcomes.

Although more data should be gathered on stage I patients, the results presented in this paper indicate that predicting the response to platin-based chemotherapy is not straightforward and more subtle than predicting advanced stage disease. Furthermore, since most published studies lack a proper independent evaluation, their results should be cautiously interpreted. We advocated the use of microarrays based on the results from our pilot study, but warned for overestimating the generalization performance, as these results were based on a cross validation technique instead of using an independent data set. Additionally, since the pilot study performance for predicting the response to platin-based chemotherapy was not statistically significant, we searched for confirmation on an independent test set. Therefore, we carried out a new study to estimate the performance of models based on independently gathered microarray data in an unbiased way. The present results, both the PCA analysis and the performance of the LS-SVM models, show that the independent evaluation is disappointing. Only the LS-SVM stage model performed well and was able to distinguish early stage and advanced stage disease on the independent data set. The PCA analysis however demonstrated that, for the three classes, the independent data did not cluster to their corresponding class in the pilot study. Additionally, the LS-SVM platin model was not able to perform better than a random predictor. Therefore, we argue that a gene expression study should be validated on independently gathered data before the results can be considered for clinical use. Independently gathered data can be influenced by subtle changes in sample preparation, sample analysis and sample hybridizations, which can deteriorate model performance. Even the techniques used by the same lab might undergo subtle changes throughout time, causing a drop in model performance when the model is applied on new patient samples. It is unclear whether published models are robust against these influences.

Additionally, ovarian cancer represents an immense variation in histological structure and biological behaviour which complicates microarray based modelling. A large number of samples is required to correctly represent the complete microscopic spectrum. It is not unlikely that an independent data set contains a different mix of tumour samples with slightly different histological characteristics compared to the pilot study, complicating independent evaluation. Moreover, the quality of the samples has a major effect on the ability to detect true differential expression and subsequent model building. However in most cases, including ours, only a limited number of samples with sufficient follow-up is available which limits our ability to obtain a similar distribution of histopathology in the pilot and independent data set, and also forces us to use archival samples instead of new ones.

The comparison of the LS-SVM stage and LS-SVM platin model with published genes sets confirmed that predicting disease stage is easier than predicting response to platin-based chemotherapy. For predicting disease stage many previously developed gene sets are able to distinguish both classes indicating that many genes change when a tumour progresses from early to advance stage disease. Predicting the response to platin based chemotherapy is more challenging. None of the previously developed gene set models related to the response to platin based chemotherapy are able to predict this outcome significantly better than chance. This indicates that these gene sets do not generalize to our independently gathered data set. Only the 27-gene model by Lancaster and colleagues [20], which distinguishes between primary ovarian cancer and metastatic tissue, is able to predict the response to platin based chemotherapy to some degree. This gene set contains 12 genes which have previously been shown to be involved in oncogenesis and 10 genes which have been implicated in the p53 pathways. The performance of this gene set on our independent data set provides some evidence that genes distinguishing between primary and metastatic tissue also play a role in resistance to therapy.

## Conclusion

Our results show that an independent evaluation of models based on gene expression data is necessary to validate models before considering subsequent steps to make microarray analysis clinically available. Previously published studies should be critically reviewed, in light of the current results, to assess if the reported model performance is not overestimated by inappropriate use of a test set and, if this is not the case, to consider if an independent study would confirm the reported model performance. Finally, prospective validation in multi-centre trials is necessary before microarray technology can move to clinical practice.

## Competing interests

The author(s) declare that they have no competing interests.

## Authors' contributions

FDS, OG, NP, FA, BDM, DT and IV conceived the study and provided clinical and mathematical background. TVG looked up patient records in the database and tissue samples in the tumour bank, performed sample annotation and gathered follow-up of patients. KE performed pre-processing of the data sets. OG, FDS and NP performed the PCA and LS-SVM analysis, and the comparison with published gene set analysis. All authors contributed to the manuscript and approved it.

## Pre-publication history

The pre-publication history for this paper can be accessed here:


